# Environmental DNA persistence and fish detection in captive sponges

**DOI:** 10.1111/1755-0998.13677

**Published:** 2022-07-12

**Authors:** Wang Cai, Lynsey R. Harper, Erika F. Neave, Peter Shum, Jamie Craggs, María Belén Arias, Ana Riesgo, Stefano Mariani

**Affiliations:** ^1^ School of Biological and Environmental Sciences Liverpool John Moores University Liverpool UK; ^2^ NatureMetrics Ltd Guildford UK; ^3^ Department of Life Sciences Natural History Museum London UK; ^4^ Horniman Museum and Gardens London UK; ^5^ School of Life Sciences University of Essex Colchester UK; ^6^ Departamento de Biodiversidad y Biología Evolutiva Museo Nacional de Ciencias Naturales (CSIC) Madrid Spain

**Keywords:** eDNA, fish biodiversity, metabarcoding, natural sampler, nsDNA, porifera

## Abstract

Large and hyperdiverse marine ecosystems pose significant challenges to biodiversity monitoring. While environmental DNA (eDNA) promises to meet many of these challenges, recent studies suggested that sponges, as “natural samplers” of eDNA, could further streamline the workflow for detecting marine vertebrates. However, beyond pilot studies demonstrating the ability of sponges to capture eDNA, little is known about the dynamics of eDNA particles in sponge tissue, and the effectiveness of the latter compared to water samples. Here, we present the results of a controlled aquarium experiment to examine the persistence and detectability of eDNA captured by three encrusting sponge species and compare the sponge's eDNA capturing ability with established water filtration techniques. Our results indicate that sponges and water samples have highly similar detectability for fish of different sizes and abundances, but different sponge species exhibit considerable variance in performance. Interestingly, one sponge appeared to mirror the eDNA degradation profile of water samples, while another sponge retained eDNA throughout the experiment. A third sponge yielded virtually no DNA sequences at all. Overall, our study suggests that some sponges will be suitable as natural samplers, while others will introduce significant problems for laboratory processing. We suggest that an initial optimization phase will be required in any future studies aiming to employ sponges for biodiversity assessment. With time, factoring in technical and natural accessibility, it is expected that specific sponge taxa may become the “chosen” natural samplers in certain habitats and regions.

## 1 INTRODUCTION

The environmental DNA (eDNA) approach is increasingly used to profile biodiversity in ecological research (Bohmann et al., 2014). This method can target multiple taxa in parallel by capturing, extracting and sequencing DNA from exfoliated cells and extracellular DNA from different environmental samples, such as water, soil, and air (Andersen et al., 2012; Eble et al., 2020; Lynggaard et al., 2022), followed by taxonomic assignment using bioinformatic tools (Cristescu, 2014). High‐throughput capability and low requirements for on‐site taxonomists mean that eDNA analysis offers considerable improvements over certain traditional survey methods (Goldberg et al., 2016; Lebuhn et al., 2013), thus research on and applications of eDNA have proliferated in the past decade (Pawlowski et al., 2020). To date, eDNA has been most extensively used to monitor aquatic biodiversity and address important ecological questions in aquatic systems (Ruppert et al., 2019).

Environmental DNA is especially beneficial to marine research, where the deployment of large‐scale and multitaxa biodiversity surveys is challenging and costly. In the marine environment, studies have typically compared eDNA performance with well‐established catch‐based and video‐based methods (Aglieri et al., 2021; Russo et al., 2021; Valdivia‐Carrillo et al., 2021). These studies generally show that eDNA is an effective and sensitive method for marine biodiversity assessment, often outperforming the traditional approaches. For instance, the eDNA approach enables a broader investigation of taxonomical diversity, facilitating the detection of elusive species (Boussarie et al., 2018), as well as proving effective at capturing more functional groups (Aglieri et al., 2021). Nevertheless, existing marine eDNA protocols are not without challenges, given the sheer size and considerable physical and ecological complexity of marine environments (Hansen et al., 2018). One limitation of marine eDNA is the sampling capacity (Goldberg et al., 2016).

Aquatic eDNA is primarily collected from the sea using water filtration via an artificial membrane (McQuillan & Robidart, 2017). Unsurprisingly, researchers have advised that large volume filtration and increased sampling replication should be considered in marine eDNA studies to avoid false negatives (Bessey et al., 2020; Stauffer et al., 2021). However, these optimizations require a substantial budget for study design (Ficetola et al., 2015) or lead to significant investment in high‐tech solutions such as integrated eDNA sampling systems (Thomas et al., 2018) and deep‐sea robotic samplers (McQuillan & Robidart, 2017). These high‐tech solutions can become limiting for small research groups in many parts of the world and studies in remote and poorly accessible environments. As an alternative to mechanical filtration systems, passive eDNA sampling has been proposed to lower technological investment via simple capture media. For example, Kirtane et al. (2020) employed adsorbent‐filled sachets to collect and preserve eDNA. Bessey et al. (2021) examined the efficiency of submerging filter membranes directly in the water column, thereby eliminating labour intensive water filtration. Another line of research, which further reduces deployment times and the use of gear and plastics, is harnessing natural eDNA samplers, i.e., live filter‐feeding organisms in aquatic ecosystems. Siegenthaler et al. (2019) used gut contents from shrimps to assess fish diversity, and Wells et al. (2021) used anemones' diet to assess plankton communities. Mariani et al. (2019) found that eDNA extracted from sponges can detect the presence of a variety of marine fish and mammals in Mediterranean and Antarctic waters. In addition, Turon et al. (2020) further highlighted the usefulness of sponges to describe tropical fish communities from coral reefs in South‐eastern Asia. This new perspective opens up possibilities for relatively low‐cost and low‐tech biodiversity monitoring. Sponges are the most efficient natural water filters on the planet (Kahn et al., 2015), and their pumping rates can vary from 0.3 to 35 ml/min/cm^3^ (Gerrodette & Flechsig, 1979; Hoffmann et al., 2008; Weisz et al., 2008). Thanks to their structure, sponges can trap objects ranging in size from microscopic particles to relatively large diatoms (Ribes et al., 1999; Riesgo et al., 2021), and have been used to uncover sponge‐associated Arthropoda and Annelida communities (Kandler et al., 2019). Given their ubiquitous distribution (Van Soest et al., 2012), regeneration ability (Ereskovsky et al., 2021), and ease of sampling, sponges have the potential to become cost‐effective natural samplers in marine ecosystems for eDNA surveys. However, sponge eDNA pumping/trapping ability is likely to be affected by many factors, such as size (Morganti et al., 2019) and symbiont content (Weisz et al., 2008), which may contribute to whether certain sponge species can serve as natural eDNA samplers.

To our knowledge, no direct comparisons of eDNA capture from sponge and water samples have been carried out yet in controlled or natural settings. Here, we designed a tank experiment to compare the persistence and detectability of eDNA between three sponge species and the standard water filtration protocol. We introduced fish species in replicated tanks with sponges for 40 h to allow sponges to accumulate eDNA, then removed the fish. Repeat sampling over a period of four days offered insights into fish eDNA degradation and detectability variance between sponges and water samples, with important implications for the future use of sponges as natural eDNA samplers.

## 2 MATERIALS AND METHODS

### 2.1 Experimental design

#### 2.1.1 Experimental facilities and materials

Aquaria experiments were completed at the Horniman Museum & Gardens (HMG), London. We set up three independent aquarium systems, each comprising a 165 L experimental tank and an 80 L filtration sump located underneath. This sump contained mechanical filtration (ClariSea SK5000 Generation 2), 3.5 kg of biological filtration (Maxspect Nano Tech Bio Spheres), and a protein skimmer (Bubble Magus Curve 9). A return pump located in the sump continuously supplied high‐quality, temperature‐controlled (*T*_mean_ = 26.8°C) seawater to the experimental tank. Water was then returned directly into the mechanical filtration of the sump via a 40 mm standpipe. Our experiments employed three sponge species: *Chondrilla* sp. (order Chondrosiida), *Axinyssa* sp. (order Suberitida), and *Darwinella* sp. (order Dendroceratida). These sponges were chosen as they had naturally colonized the coral colonies at the HMG aquarium facilities, and because of their different filtering characteristics. *Chondrilla* sp. has a slower pumping rate than *Axinyssa* sp. and *Darwinella* sp., and is known as a high microbial abundance (HMA) sponge type (Batista et al., 2018; Díez‐Vives et al., 2020; Moitinho‐Silva et al., 2017). These colonizing sponges at the aquarium facilities offered an ideal head start for our comparative experiment, as it is typically difficult to de novo rear sponges with different filtration characteristics simultaneously in artificial environments.

**FIGURE 1:**
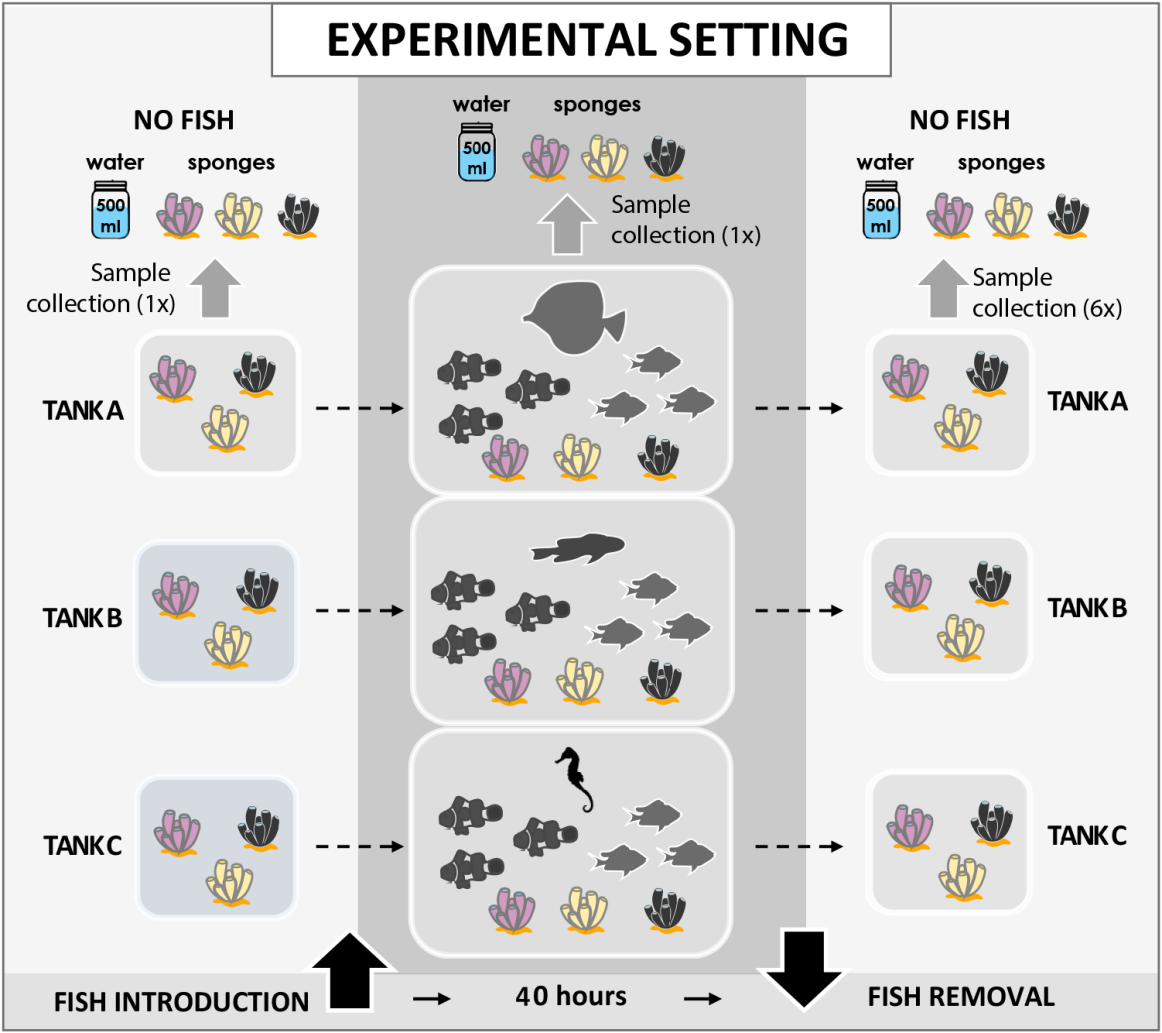
Experimental setting. There are three main phases of the experiment. The first phase took place prior to introducing fish into tanks, when there were only sponges. The second phase involved placing fish into the experimental tanks for 40 h. In the last phase, fish were removed and sponges remained in tanks for 72 h. Samples were collected at six time points: 0, 4, 8, 24, 48, and 72 h after removing fish in the last phase

As shown in Figure 1, before being placed into the experimental tanks, the sponges were quarantined in a separate empty tank (with no fish) for seven days to ensure that fish eDNA would be removed from their tissue prior to the subsequent experiments. Following an acclimation period after sponges were placed in the experimental tanks, both water and sponge samples were collected to provide a detection baseline. Five fish species were employed in replicated tanks. Three individuals of clown anemonefish (*Amphiprion ocellaris*) and three individuals of blue‐green damselfish (*Chromis viridis*) were placed into each tank. We placed one individual of sailfin tang (*Zebrasoma veliferum*) into tank A, one individual of royal gramma (*Gramma loreto*) into tank B, and one individual of half‐spined seahorse (*Hippocampus semispinosus*) into tank C. Each tank contained the same three species of sponges, two fish species common across the three tanks, and one fish species unique to each tank.

#### 2.1.2 DNA degradation experiment

Sponges were allowed to acclimate to experimental tanks for three days. Fish were introduced and remained in the tanks unfed for 40 h, then all fish were removed while sponges remained in the tanks for another 72 h. Water and sponge samples were collected at eight time points (Figure 1): one sample was taken before fish introduction (considering the possibility that sponges still contained or introduced eDNA from nonexperimental fish), one sample was taken when fish had been in the tanks for 20 h (considering the possibilities that eDNA may already be present in the water column and sponge tissue or that eDNA may not yet be detectable in water or sponges), then samples were taken at 0, 4, 8, 24, 48, and 72 h after fish removal.

For each time point in each tank, we collected one water sample and three sponge samples (one biopsy for each species), totalling 96 samples (4 samples × 8 time points × 3 tanks). A 500 ml water sample was collected and filtered through a 0.45 μm Sterivex filter (PES membrane, Merck Millipore) using a 60 ml syringe (Fisher Scientific). Each filter was placed into a single bag and then immediately stored at −20°C until DNA extraction. Using arm‐length gloves with wrist gloves on top, sponge biopsies were taken using disposable scalpels (Swann‐Morton no. 21) and stored in 2 ml tubes with 100% ethanol at −20°C. At the end of the experiment, all sponge biopsies were transferred into fresh 2 ml tubes with 100% ethanol and stored at −20°C until DNA extraction. The sponge biopsies samples (<0.5 cm^3^, ≤50 mg) were much smaller than the whole individual (~10 cm^3^) to minimize stress on the organism.

In addition, we included one seawater blank from the filtration system (500 ml), one filtration blank (500 ml of MilliQ water), and one sponge blank (an artificial kitchen sponge submerged in a sterile 10 L plastic box containing seawater from the filtration system) on each experimental day to assess for potential contamination. All sampling implements (water bottle, tubes, scalpel, long glove, and laboratory materials) were sterilized prior to and disposed of after sample collection. Between sampling events, workspaces were decontaminated using 10% v/v bleach solution (made from Cleanline thin bleach containing 4.53% sodium hypochlorite) followed by 70% v/v ethanol solution.

### 2.2 Laboratory procedures

Each filter capsule was opened onto a Petri dish using carpenter pliers. The filters were removed from the inner tube and torn into small pieces using metal forceps. All pieces were placed inside a 1.5 ml microtube for DNA extraction. For sponge biopsies, approximately 25 mg of dry sponge per sample was used for DNA extraction. Each sponge was removed from the storage ethanol, blotted dry against filter paper (42.5 mm, Fisher Scientific) inside a Petri dish, then placed inside a 1.5 ml microtube for DNA extraction. All samples were processed using the Mu‐DNA tissue protocol with an inhibitor removal step from the Mu‐DNA water protocol (Sellers et al., 2018). The extracts were then quantified for DNA concentration using a Qubit 4 fluorometer with a Qubit dsDNA HS Assay kit (Thermo Fisher Scientific). Thirty‐nine out of 72 sponge DNA extracts were diluted 1:10 to enable PCR amplification. Aquatic eDNA extracts were not diluted for PCR amplification due to their low DNA concentration.

All reusable equipment for DNA extraction was first sterilized in 10% v/v bleach solution, followed by a rinse in 5% v/v lipsol detergent and deionized water. Equipment and consumables were then exposed to 30 minutes of ultraviolet (UV) light.

PCR amplifications were carried out using the Tele02 primers (Taberlet et al., 2018), which amplify a ~169 bp fragment of the mitochondrial 12S rRNA gene. Primers contained unique 8‐bp dual barcodes for sample identification and to reduce tag jumping (Schnell et al., 2015), with 2–4 leading “N” bases to increase sequence diversity. Samples were amplified in triplicate under the following conditions: initial denaturation at 95°C for 10 min, followed by 40 cycles of 95°C for 30 s, 60°C for 45 s, 72°C for 30 s, and finishing at 72°C for 5 min. All PCRs were performed in 20 μl reactions containing 10 μl 2× MyFi Mix (Meridian Bioscience), 0.5 μM of each primer, 0.04 mg BSA (Bovine Serum Albumin Solution, Thermo Fisher Scientific), 5.84 μl of molecular grade water (Invitrogen), and 2 μl of DNA template. Two or three PCR positive and negative controls were included on each PCR run—two of each for eDNA samples and three of each for natural sampler DNA samples, given the higher overall number of nsDNA samples. PCR positive controls (0.05 ng/μl) contained one fish species which was not present in the HMG (the iridescent catfish *Pangasianodon hypophthalmus*). PCR triplicates were pooled together and visualized on 2% agarose gels. Each sample was purified using Mag‐Bind Total Pure NGS (Omega Bio‐Tek) magnetic beads. Purified PCR products were quantified as above and pooled in equimolar amounts to create two libraries, one for eDNA samples (24 samples and 18 controls), and one for nsDNA samples (72 samples and 16 controls). Pooled PCR products were purified with magnetic beads, and libraries were prepared using the NEXTFLEX Rapid DNA‐Seq Kit for Illumina (PerkinElmer) following the manufacturer's instructions. The libraries were quantified by quantitative PCR (qPCR) using the NEBNext Library Quant Kit for Illumina (New England Biolabs) and fragment size was checked using the Tape Station 4200 (Agilent). They were then pooled in equimolar concentrations with a final molarity of 60 pM with 10% PhiX control. The libraries were sequenced on an Illumina iSeq100 using iSeq i1 Reagent version 2 (300 cycles) at Liverpool John Moores University.

### 2.3 Bioinformatic and statistical analysis

The bioinformatic processing was based on the OBITools software 1.2.11 (Boyer et al., 2016). The raw sequencing data were first trimmed to remove low‐quality ends using ‘obicut’. After trimming, paired‐end reads were merged by ‘illuminapairedend’, and alignments with low (<40) quality scores were removed. The alignments were then demultiplexed using ‘ngsfilter’ with default parameters. Subsequently, quality filters were performed by ‘obigrep’ to retain sequences between 130 and 190 bp without ambiguity to filter out erroneous sequences, followed by dereplication using ‘obiuniq’, and chimera removal using the de novo chimera search function in vsearch 2.4.3 (Rognes et al., 2016). The remaining sequences were clustered by swarm version 2.1.3 (Mahé et al., 2015) with “‐d 3”. Taxonomic assignment was performed via ‘ecotag’. The reference database used in ecotag was constructed by in silico PCR for Tele02 primers against the EMBL database (Release version r143) using ‘ecoPCR’. An additional taxonomic assignment was carried out using BLAST against the NCBI reference database to check the assignment of sequences, and we manually corrected one species (*Gramma loreto*) that ecotag could only assign to the metazoan level. Finally, a sample/OTU table with taxon information was formatted using R scripts listed at https://github.com/metabarpark/R_scripts_metabarpark. We then used the R package lulu 0.1.0 (Frøslev et al., 2017) with default parameters to filter erroneous OTUs based on the calculation of OTUs' pairwise similarities and co‐occurrence patterns. The remaining OTUs were further collapsed using the metabarpark owi_collapse R script.

All downstream statistical analyses were carried out in R version 3.6.3. The raw read counts were first transformed to log_10_ read counts for visualizing the relationship between time and the read counts of each sample, and we kept read count zero instead of infinity (log_10_(0)). We also carried out the same analysis as using log_10_ transformed reads by using other data transformations (relative abundance), and the results can be found in the Supporting Information. To examine the relationship between time and transformed reads or species richness, we performed linear models (LMs) using the “lm” function. We visualized community composition by the pheatmap function (version 1.0.12; Kolde, 2019). The transformed read counts then were converted to presence/absence (1/0) for subsequent analyses. To compare pairwise differences in species richness between samples, we performed multiple pairwise post hoc comparisons using the TukeyHSD function on the ANOVA object. Subsequently, we used mvabund version 3.12.3 (Wang et al., 2012) to analyse the effects of covariates (sample type, time, and tank) on community composition. Mvabund is a model‐based method (generalized linear model framework) which allows us to select an appropriate error distribution for the corresponding data type. Here we used the manyglm function with binomial error distribution for the presence/absence data to fit glms for each species and then used the “anova.manyglm” function to indicate the treatment effect. All results were visualized using the ggplot2 R package version 3.3.5.

## 3 RESULTS

### 3.1 Bioinformatic processing and taxonomic composition

Sequencing yielded nearly 1 million raw sequences, including 703,816 for the sponge natural sampler DNA library and 295,854 for the aquatic eDNA library. After quality filtering, we retained 652,148 paired‐end reads, clustered into 175 OTUs. OTUs were assigned to experimental fish species, PCR positive control species, human, and nonexperimental fish species present in the HMG facilities and in feeds. A total of 119 OTUs (8.6% of read counts) could not be identified below the class level (Table S1). Three experimental fish species were assigned to the species level, and two were assigned to the genus‐level with high confidence (100%). Five OTUs were assigned to nonexperimental fish species (genus‐level, >97% identity). These nonexperimental species were probably carried over to the experimental setup by the sponges, as most were detected at the beginning of the experiment (Figure S2; day one, sponge samples). We then chose a stringent level to minimize false positives and contamination by removing low‐abundance read counts of OTUs (10 ≤ reads), according to the highest read counts of nonpositive control species and the lowest read counts of true positive species in PCR positive controls. After filtering, the control blanks did not contain fish sequences, indicating that sampling and decontamination procedures were appropriate and false positives/contamination would not affect downstream analysis. In addition, the non‐experimental fish OTUs detected in the corresponding tanks were removed based on the samples collected before fish introduction. The details of species composition are shown in Figures S2 and S3. For downstream analysis, we only considered the five experimental fish species.

**FIGURE 2:**
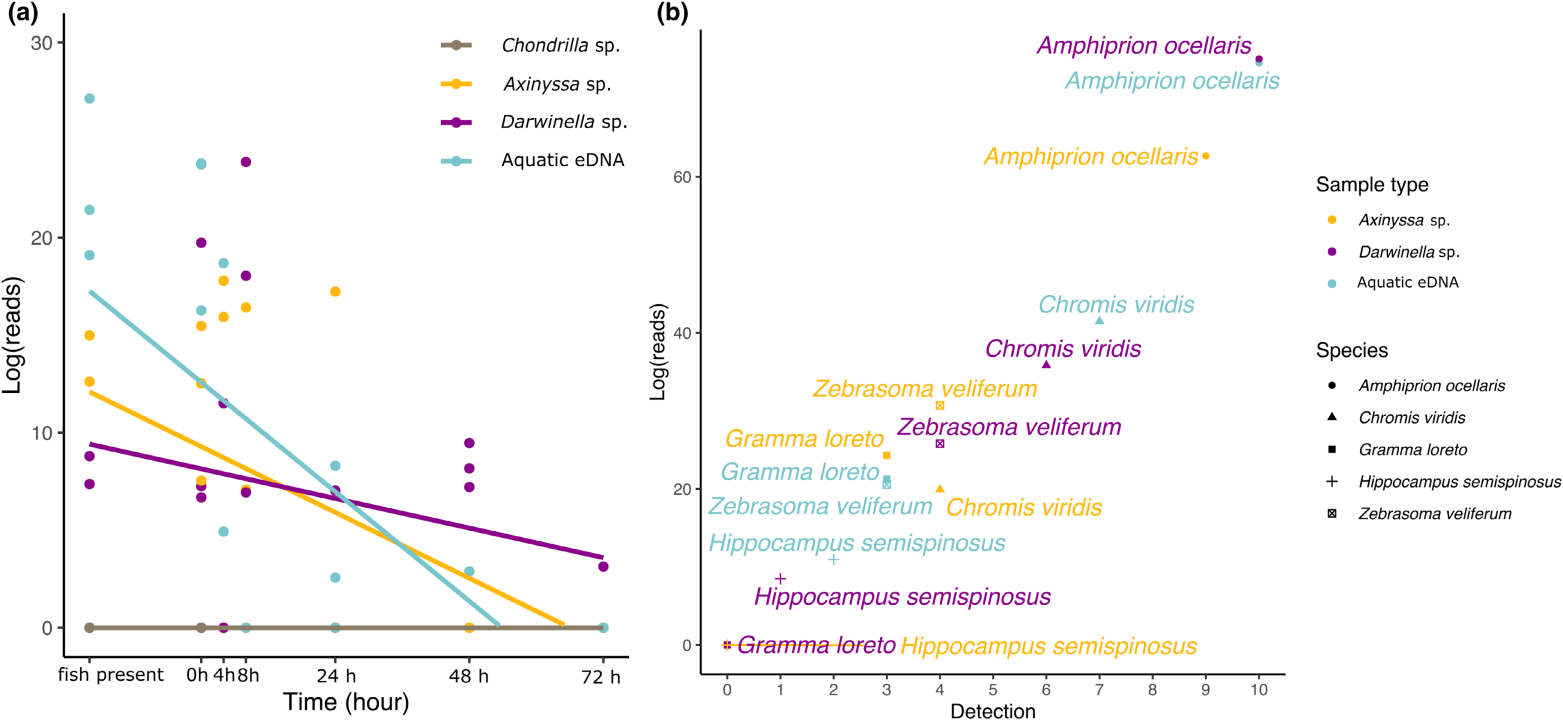
Comparing the total read counts of each sample and the detectability of each species. (a) the linear relationship between the total log_10_ read counts and the time point of each sample type. The points represent sample replicates per tank. The x‐axis is the timeline of the sampling events. The “fish present” represents the period when fish were in the tanks, the time represents the time point after fish removal. (b) the detectability of each fish species per sample type. Symbols represent species. The y‐axis is the total log_10_ read counts of each species per sample type, and the x‐axis is the total number of positive detections of each fish per sample type. The colours represent the sample type for both a and b

The total transformed reads for the five experimental species decreased across samples over time (Figure 2a, also see Table S2 for entire linear model output) but varied among samples. There was a significant negative relationship between the total transformed reads and time for the aquatic eDNA samples (*p* < .001, *R* ^2^ = .443) and the sponge *Axinyssa* sp. (*p* = .009, *R* ^2^ = .276), while the total transformed reads of *Darwinella* sp. showed no correlation with time (*p* = .233, *R* ^2^ = .025). *Chondrilla* sp. did not show any positive detections over time.

### 3.2 The detectability of each species across nsDNA and eDNA

To compare the detection efficiency of different fish between sponge nsDNA and aquatic eDNA, we examined the detection rate and transformed reads per species for each sample (Figure 2b). Overall, detection frequency was correlated with read abundance, but varied considerably among the experimental species. The abundant species *Amphiprion ocellaris* and *Chromis viridis* (three individuals in each tank) had higher detection rates than *Z. veliferum*, *G. loreto* and *H. semispinosus* (one individual in each tank); however, *A. ocellaris* and *C. viridis* also differed from each other. Furthermore, the relatively larger individual (*Z. veliferum*) had a higher detection rate than the other two smaller, unique fishes. This pattern was consistent across aquatic eDNA and sponge nsDNA samples (Figure 2b).

### 3.3 Degradation of nsDNA and eDNA

**FIGURE 3:**
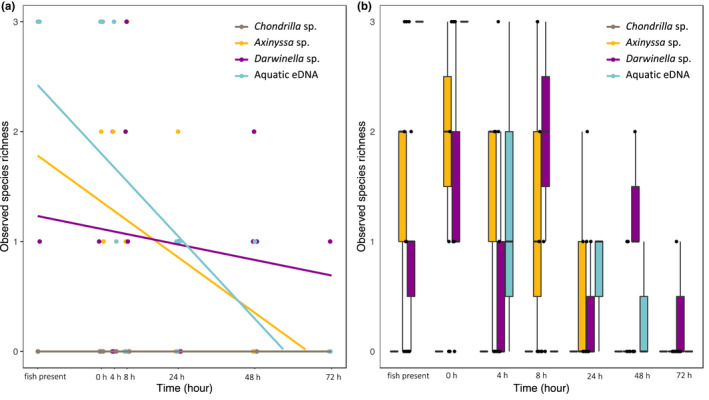
Comparison of alpha diversity among sample types. Read count data was converted to presence/absence data, thus, observed species richness was calculated using species incidence. The highest observed species richness is 3, indicating the sample can detect all fish. On the contrary, 0 indicates that no species are detected. (a) the linear relationship between alpha diversity and time. (b) Summary of species richness of each sample type at each time point. Colours and time code as in Figure 2a

The linear model analysis found significantly different decay patterns between sponge nsDNA and aquatic eDNA (Figure 3, also see Table S3 for entire linear model output). We used presence/absence data, focusing on species richness rather than read abundance. Species richness declined steeply over time in water samples (*p* < .001, *R* ^2^ = .437), and also significantly, albeit less steeply, in the sponge *Axinyssa* sp. (*p* = .008, *R* ^2^ = .278), while *Darwinella* sp. showed no significant decrease over the duration of the experiment (*p* = .424, *R* ^2^ = −.017, Figure 3b). Time effect aside, there was no significant difference in species richness between aquatic eDNA and sponge nsDNA during the observation period (Table S4: Tukey's comparisons, *p* > .5; omitting *Chondrilla* sp. which failed to amplify).

**FIGURE 4:**
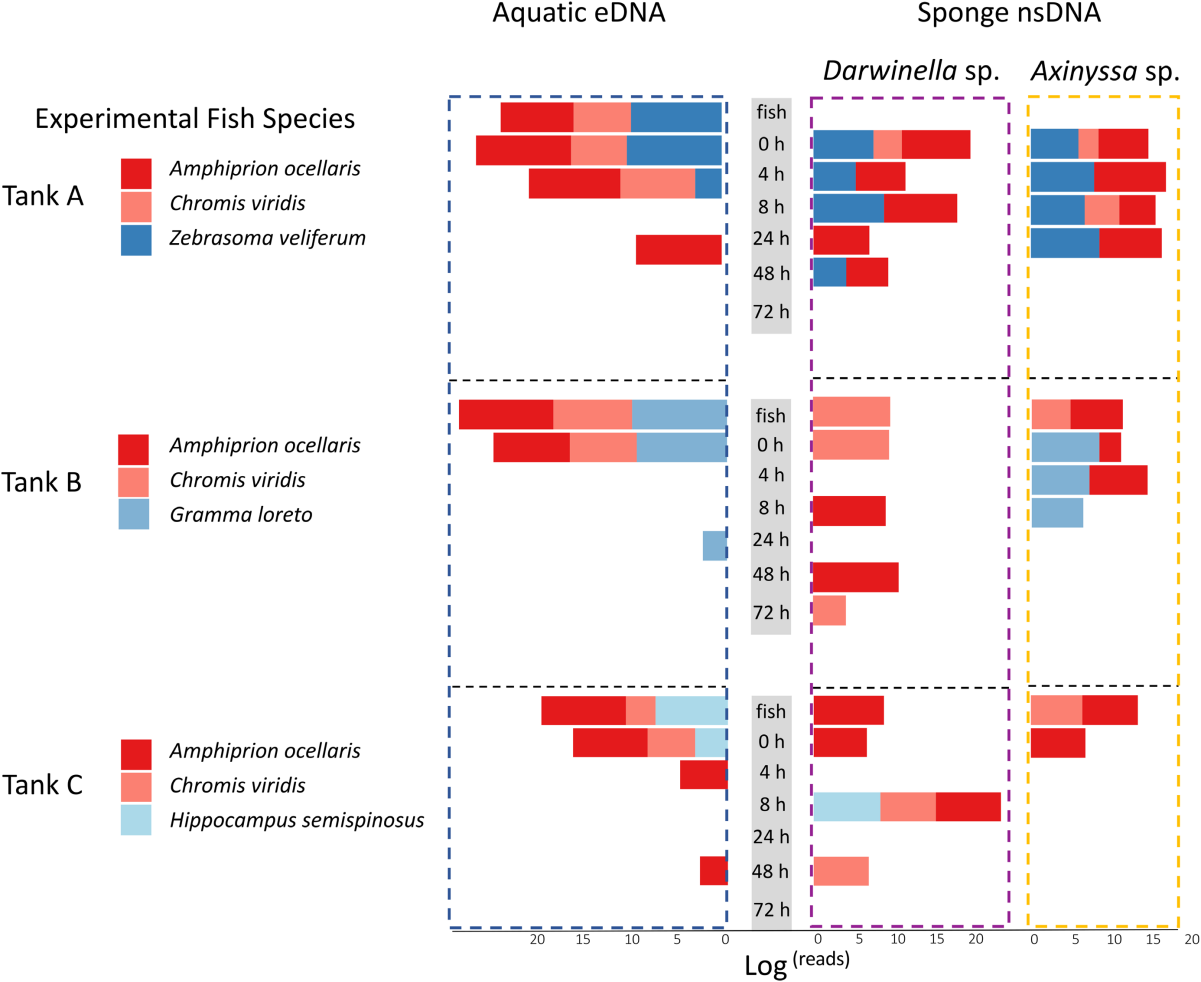
Community change over time. The horizontal panels represent three tanks; the vertical panels show three sample types. Water and sponge samples are separated by the timeline. For each tank, the community changes from top to bottom according to sampling time, and time codes as in Figure 2a. The length of the bar is based on log_10_ read counts. When there is no bar at the corresponding time point, it represents that no fish species were detected. Colour codes refer to the experimental fish species for each tank (three species for each tank, two of which ubiquitous)

Changes in fish community composition for aquatic eDNA and sponge nsDNA over time across tanks can be seen in Figure 4. The overall communities detected during the observation period did not significantly differ among sample types (mvabund*: p* = .70, *df* = 2), but community change was detected over time and more obviously in aquatic eDNA than in sponge nsDNA (Table S5). During the first 40 h (when fish were present in the tanks), aquatic eDNA consistently detected the entire community across the three tanks, while sponge nsDNA appeared to be less efficient in capturing the whole fish community (Figure 4). However, after 4 h of removing fish, aquatic eDNA degraded rapidly, and a drop in species detection with the aquatic eDNA was observed. On the contrary, fish community change was less notable in sponge nsDNA throughout the experimental period, especially in *Darwinella* sp. (*p* = .852, *df* = 1). This result is consistent with the species richness pattern observed (see Figure 3). Sponge nsDNA failed to detect the entire fish community in a single sampling event but detected fish over longer periods of time than aquatic eDNA (Figures 3 and 4).

## 4 DISCUSSION

Our study confirms general expectations that sponges capture eDNA from the surrounding environment and emphasizes the variance in eDNA persistence and detectability among different sponge species. We employed three sponge species based on their established presence at the experimental facilities, and each one performed in a very distinct manner.

A key finding of our study was that fish species present in greater abundance or of larger size are more likely to be detected and contribute more reads to natural sampler DNA (Figure 2b). This suggests that those sponges that are suitable for natural eDNA sampling might also be used to infer the relative abundance of fish. Future studies in more diverse wild ecosystems should assess the degree to which nsDNA concentration is correlated with species abundance or size, although the use of metabarcoding read counts to estimate relative species abundance still requires much ground‐truthing (Cristescu & Hebert, 2018). Despite the simplified fish community used in this study, without choosing any particular sponge species with well‐studied morphology and filtration characteristics, it is reassuring to see that at least some sponges are comparable with aquatic eDNA filtration, which is a powerful tool for monitoring fish densities (Levi et al., 2019), and abundance/biomass (Carvalho et al., 2022; Di Muri et al., 2020), leading to the expectation that sponges could indeed be adopted for such applications in the future. Further sponge nsDNA‐based studies could also be carried out to examine the quantitative methods developed for aquatic eDNA, such as qPCR and droplet digital PCR using species‐specific primers (Baker et al., 2018; Levi et al., 2019).

Another primary goal of this study was to investigate eDNA decay in sponges compared to water samples. Our results suggest that eDNA decay rates differ in sponge nsDNA and aquatic eDNA samples. For aquatic eDNA, as soon as the fish were removed from the tanks, the eDNA degraded rapidly over a 4‐h period; after which fish species were poorly detected (Figure 4). In contrast, fish can be detected for longer periods in sponge nsDNA samples; especially in *Darwinella* sp., where eDNA did not degrade dramatically over time, at least over the short time frame of the experiment (72 h). This is likely because these sponges are moderately effective pumpers, trapping eDNA efficiently and aided by a slow metabolism (Morganti et al., 2019) and low microbial abundance (Moitinho‐Silva et al., 2017), allowing them to preserve eDNA over longer periods. In the case of delayed detection of very low eDNA concentrations, it might take time for a sponge to accumulate eDNA in its tissue to a detectable level. However, due to the interaction between sponge physiology and symbiont content (Ribes et al., 1999; Hentschel et al., 2006; Leys & Hill, 2012), it is possible that some sponges may have faster eDNA decay rates than aquatic eDNA in open water. Here our empirical evidence shows that some sponges will preserve DNA for longer than eDNA persists in water. It is possible to see how this feature could also be beneficial in monitoring rare migratory species from remote areas.

As demonstrated by previous studies (Mariani et al., 2019; Turon et al., 2020), sponge nsDNA can detect pelagic or migratory species visiting some locations infrequently. Our evidence further demonstrates how certain sponges could help monitor these more elusive species (preserving eDNA for longer), which other monitoring methods may miss. As for the resident species, sponge nsDNA may provide insightful fish detection compared to aquatic eDNA because spatial and temporal variability of aquatic eDNA may affect species detection (Allan et al., 2021; Canals et al., 2021). Thus, the sessile nature of sponges could more exhaustively track the diel fluctuations and other behaviours of fishes. However, the collection of these effective natural samplers is partly limited by sampling requirements. In some shallow coastal waters, sponges will be accessible through simple wading and snorkelling activities, while in other circumstances scuba‐diving would be required, and divers should have some familiarity with sponge morphology and taxonomy. Despite these limitations, sponge sampling could be conducted at the same time as studies investigating fish diversity using underwater visual census (which also requires diving), or sponges that have been collected for other research purposes can be reused or subsampled for metabarcoding. Furthermore, some sponge species typically settle on various human‐made structures (e.g., piers, moorings, and oil rigs) and colonize artificial reefs. These sponges are easily collected and are ideal candidates for linking biodiversity assessment with human impact (Wulff, 2001; Vad et al., 2021).

The natural sampler approach is in its infancy and requires much validation in natural scenarios. Little is known about the influence of many factors that affect eDNA degradation rates and transport (Collins et al., 2018; Holman et al., 2021; Strickler et al., 2015) inside sponge tissues. Morganti et al. (2019) showed that temperature has little effect on sponge pumping rate, while the size of the sponge is the primary determinant. Knowledge of the pumping rate of target sponges may therefore be of key importance for successful nsDNA approaches, as should be some level of understanding of the influence of microbial symbionts.

Nevertheless, biotic processes, mostly linked to morphology, filtration rate, physiology, and symbiosis, necessitate further research. In our study, *Chondrilla* sp. failed to detect any fish. This could be due to the intrinsic inability of this species to capture or preserve eDNA. Members of the genus *Chondrilla* are high microbial abundance (HMA) sponges, compared to their counterparts of the genera *Axinyssa* and *Darwinella* (Batista et al., 2018; Díez‐Vives et al., 2020; Moitinho‐Silva et al., 2017). The substantial symbiotic load of HMA sponges could slow filtration and accelerate metabolism, thus influencing the capture and persistence of eDNA. HMA sponges filter 30%–40% slower than low microbial abundance (LMA) sponges (Weisz et al., 2008), even though chondrosiids are relatively effective pumpers (Milanese et al., 2003). Therefore, *Chondrilla* sp. is at a disadvantage in the competition for eDNA capture in tanks compared to the LMA sponges (*Axinyssa* sp. and *Darwinella* sp.). Furthermore, exposure to manipulative stress, such as those in our experiment, may also affect filtration characteristics, while PCR inhibitors, such as pigments and natural metabolites, or even an aggressive microbiome, may all play a part in eDNA detection from sponge tissue. Therefore, collecting several sponge species from the same habitat could also serve as a good strategy for maximizing taxon detection in natural settings. A previous study has also shown that not all sponges have the same ability to capture and retain eDNA, but sponge morphology did not significantly affect the detected OTU richness (Turon et al., 2020). However, similar comparisons have yet to be made in different habitats. Increasing sampling effort, wherever it does not damage sponge communities, may improve the detection of fish species in an area, especially in hyper‐diverse environments.

We note that the sponge biopsies used in our experiment may be insufficient to capture biodiversity in natural environments. We were constrained by the need to keep the sponges alive and pumping for the duration of the experiment, hence minimizing physical damage; but it is reasonable to suggest that, in natural biomonitoring contexts, collecting several pieces of tissues from large sponges and/or, where plentiful, sacrificing entire individuals will minimize the risk of false negatives. Overall, a deeper understanding of the underlying mechanism of how sponges capture and preserve eDNA will be helpful for users to design different applications.

## 5 CONCLUSION

While we are still far from a standardized workflow for sponge natural sampler DNA, we can identify a number of directions towards that goal. Our study focused on comparing eDNA decay between sponge and water samples, but much work is still needed to optimize tissue preparation for DNA isolation, determine the size/quantity of sponge tissue required, evaluate the effects of various DNA extraction and PCR strategies that will influence eDNA detection, and the biological and technical replication required for estimating biodiversity (Bohmann et al., 2021; Kumar et al., 2020). It will also be essential to ground‐truth the performance of sponge nsDNA in multiple natural settings, using a variety of sponge species, in comparison with aquatic eDNA samples and established conventional methods. Once these outstanding challenges are met, sponge nsDNA will offer the advantages of a cost‐effective method for the detection of fish diversity that is comparable to that of aquatic eDNA, which could significantly streamline sampling operations, reduce the use of plastic, and perhaps provide a number of biological features that will significantly enhance the toolkit of marine eDNA to further bolster biodiversity assessment.

## AUTHOR CONTRIBUTIONS

Stefano Mariani, Ana Riesgo and Lynsey R. Harper designed the study, Lynsey R. Harper, Jamie Craggs, María Belén Arias and Erika F. Neave handled specimens and collected samples, Lynsey R. Harper and Erika F. Neave performed the molecular experiments with assistance from Peter Shum; Wang Cai, and Peter Shum performed the bioinformatic analyses. Wang Cai performed the statistical analyses and wrote the first draft of the manuscript, and all authors contributed substantially to interpretations and revisions.

### OPEN RESEARCH BADGES

This article has earned an Open Data badge for making publicly available the digitally‐shareable data necessary to reproduce the reported results. The data is available at https://doi.org/10.5281/zenodo.6603365.

## Supporting information


Appendix S1

## Data Availability

Raw sequencing data and all bioinformatics and statistical analysis files are deposited in the Zenodo. The archive is available for downloading at: https://doi.org/10.5281/zenodo.6603365 All collaborators are included as coauthors.
