# Supplementary material for: Environmental DNA persistence and fish detection in captive sponges
Source: Mol Ecol Resour. 2022 Jul 12;22(8):2956–66. doi: 10.1111/1755-0998.13677 (PMC9796769; doi:10.1111/1755-0998.13677)
Supplement: Supplementary file 1 — Appendix S1 [file MEN-22-2956-s001.docx]

# Title: Environmental DNA persistence and fish detection in captive sponges

**Running title:** sponge nsDNA versus aquatic eDNA performance

**Appendix S1:** Figures S1-S3; Table S1-S5

**Appendix S2:** Figures S4-S5; Table S6

**Authors:**

Wang Cai^1†^, Lynsey R. Harper^1,2†^, Erika F. Neave^1,3†^, Peter Shum^1^, Jamie Craggs^4^, María Belén Arias^3,5^, Ana Riesgo^3,6^, Stefano Mariani^1*^

**Author affiliations:**

^1^ School of Biological & Environmental Sciences, Liverpool John Moores University, Liverpool L3 3AF, UK

^2^ NatureMetrics Ltd, 1 Occam Court, Surrey Research Park, Guildford, GU2 7HJ, UK

^3^ Department of Life Sciences, Natural History Museum, Cromwell Road, South Kensington, London SW7 5BD, UK

^4^ Horniman Museum and Gardens, 100 London Road, Forest Hill, London, SE23 3PQ, UK

^5^ School of Life Sciences, University of Essex, Colchester Campus, CO4 3SQ, UK

^6^ Departamento de Biodiversidad y Biología Evolutiva, Museo Nacional de Ciencias Naturales (CSIC), Calle José Gutiérrez Abascal 2, 28006, Madrid, Spain

**Appendix S1:**

**Figure S1. Sponge species used in our experiment.**

**Figure S2. Taxonomic composition heatmap of controls before (A) and after (B) filtering low-abundance reads.** The figure presents the log-read counts of species/OTUs of each control.

**A.**

B.

**Figure S3. Taxonomic composition heatmap of samples before (A) and after (B) filtering of low-abundance reads.** The figure presents the log-read counts of species/OTUs per sample.

A.

****B.

**Table S1. Taxonomic assignment of sequencing data.**

| **Assignment** | **Level** | **Read counts** | **Percentage** |
| --- | --- | --- | --- |
|  |  | 652148 | 100.000% |
| *Pangasianodon hypophthalmus* | species | 191504 | 29.365% |
| *Homo sapiens* | species | 136193 | 20.884% |
| *Amphiprion ocellaris* | species | 103649 | 15.893% |
| *Zebrasoma veliferum* | species | 57040 | 8.746% |
| *Gramma loreto* | species | 38099 | 5.842% |
| *Pomadasys* sp. | genus | 21269 | 3.261% |
| *Chromis viridis* | species | 13802 | 2.116% |
| *Brevoortia tyrannus* | species | 9920 | 1.521% |
| *Mallotus villosus* | species | 9091 | 1.394% |
| *Hippocampus semispinosis* | species | 6559 | 1.006% |
| Phasianidae | family | 5316 | 0.815% |
| Astropectinidae | family | 2317 | 0.355% |
| *Siganus* sp. | genus | 631 | 0.097% |
| *Odonus niger* | species | 415 | 0.064% |
| *Halichoeres* sp. | genus | 35 | 0.005% |
| Unidentified Metazoa | kingdom | 56308 | 8.634% |

**Table S2. Linear model output**. Testing for the relationship between transformed read counts and time.

| nsDNA *Darwinella* sp. | |  |  |  |  |
| --- | --- | --- | --- | --- | --- |
|  | Estimate | Std. Error | t value | Pr(>\|t\|) | signif. |
| (Intercept) | 27.514 | 9.173 | 2.999 | 0.00737 | ** |
| reads | -1.169 | 0.949 | -1.232 | 0.23292 |  |
| Residual standard error: 29.38 on 19 degrees of freedom | | | | | |
| Multiple R-squared:  0.07399, Adjusted R-squared:  0.2598 | | | | | |
| F-statistic: 1.518 on 1 and 19 DF, p-value: 0.2329 | | | | | |
|  |  |  |  |  |  |
| nsDNA *Axinyssa* sp. | |  |  |  |  |
|  | Estimate | Std. Error | t value | Pr(>\|t\|) | signif. |
| (Intercept) | 33.9582 | 7.4212 | 4.576 | 0.000206 | *** |
| reads | -2.2176 | 0.7559 | -2.933 | 0.008527 | ** |
| Residual standard error: 25.33 on 19 degrees of freedom | | | | | |
| Multiple R-squared: 0.3117, Adjusted R-squared: 0.2755 | | | | | |
| F-statistic: 8.605 on 1 and 19 DF, p-value: 0.008527 | | | | | |
|  |  |  |  |  |  |
| Aquatic eDNA | |  |  |  |  |
|  | Estimate | Std. Error | t value | Pr(>\|t\|) | signif. |
| (Intercept) | 35.6257 | 6.2435 | 5.706 | 1.68E-05 | *** |
| reads | -2.0145 | 0.4898 | -4.113 | 0.000592 | *** |
| Residual standard error: 22.2 on 19 degrees of freedom | | | | | |
| Multiple R-squared: 0.471, Adjusted R-squared: 0.4432 | | | | | |
| F-statistic: 16.92 on 1 and 19 DF, p-value: 0.0005919 | | | | | |

Signif. codes: 0 ‘***’ 0.001 ‘**’ 0.01 ‘*’ 0.05 ‘.’ 0.1 ‘ ’ 1

| Aquatic eDNA | |  |  |  |  |
| --- | --- | --- | --- | --- | --- |
|  | Estimate | Std. Error | t value | Pr(>\|t\|) | signif. |
| (Intercept) | 1.798089 | 0.268851 | 6.688 | 2.15E-06 | *** |
| Time | -0.031274 | 0.007695 | -4.064 | 0.000662 | *** |
| Residual standard error: 1.024 on 19 degrees of freedom | | | | | |
| Multiple R-squared: 0.465, Adjusted R-squared: 0.4369 | | | | | |
| F-statistic: 16.52 on 1 and 19 DF, p-value: 0.000662 | | | | | |
|  |  |  |  |  |  |
| nsDNA *Darwinella* sp. | |  |  |  |  |
|  | Estimate | Std. Error | t value | Pr(>\|t\|) | signif. |
| (Intercept) | 1.114123 | 0.251185 | 4.435 | 0.000284 | *** |
| Time | -0.005874 | 0.00719 | -0.817 | 0.424055 |  |
| Residual standard error: 0.9567 on 19 degrees of freedom | | | | | |
| Multiple R-squared: 0.03394, Adjusted R-squared: -0.01691 | | | | | |
| F-statistic: 0.6675 on 1 and 19 DF, p-value: 0.4241 | | | | | |
|  |  |  |  |  |  |
| nsDNA *Axinyssa* sp. | |  |  |  |  |
|  | Estimate | Std. Error | t value | Pr(>\|t\|) | signif. |
| (Intercept) | 1.361219 | 0.249178 | 5.463 | 2.86^e^-05 | *** |
| Time | -0.021043 | 0.007132 | -2.95 | 0.00821 | ** |
| Residual standard error: 0.949 on 19 degrees of freedom | | | | | |
| Multiple R-squared: 0.3142, Adjusted R-squared: 0.2781 | | | | | |
| F-statistic: 8.705 on 1 and 19 DF, p-value: 0.008214 | | | | | |

**Table S3. Linear model output**. Testing for the relationship between species richness and time.

Signif. codes: 0 ‘***’ 0.001 ‘**’ 0.01 ‘*’ 0.05 ‘.’ 0.1 ‘ ’ 1

**Table S4.** TukeyHSD for comparing species richness among sample types.

|  |  | |  | |  |  |
| --- | --- | --- | --- | --- | --- | --- |
|  | diff | lwr | | upr | | p_adj_ |
| *Darwinella* sp. - *Axinyssa* sp. | 0.0476 | -0.8097 | | 0.9050 | | 0.9902 |
| WATER- *Axinyssa* sp. | 0.2380 | -0.6193 | | 1.0955 | | 0.7832 |
| WATER- *Darwinella* sp. | 0.1904 | -0.6669 | | 1.0479 | | 0.8550 |

**Table S5. Mvabund analysis**. Testing for the effects of sample type, time, tank, and their interaction on community composition.

| Aquatic eDNA + Sponge nsDNA | |  |  |  |  |
| --- | --- | --- | --- | --- | --- |
|  | Res.Df | Df.diff | score | Pr(>score) | Signif. |
| (Intercept) | 62 |  |  |  |  |
| Sample type | 60 | 2 | 5.62 | 0.697 |  |
| Time | 59 | 1 | 12.69 | 0.004 | ** |
| Tank | 57 | 2 | 38.48 | 0.001 | *** |
| Sample type:Time | 55 | 2 | 8.77 | 0.180 |  |
| Sample type:Tank | 51 | 4 | 3.01 | 0.932 |  |
| Time:Tank | 49 | 2 | 1.33 | 0.832 |  |
| Sample type:Time:Tank | 45 | 4 | 7.91 | 0.081 | . |
|  |  |  |  |  |  |
| Aquatic eDNA |  |  |  |  |  |
|  | Res.Df | Df.diff | score | Pr(>score) | Signif. |
| (Intercept) | 20 |  |  |  |  |
| Time | 19 | 1 | 12.304 | 0.004 | ** |
| Tank | 17 | 2 | 21.912 | 0.002 | ** |
| Time:Tank | 15 | 2 | 1.645 | 0.657 |  |
|  |  |  |  |  |  |
| nsDNA *Axinyssa* sp. |  |  |  |  |  |
|  | Res.Df | Df.diff | score | Pr(>score) | Signif. |
| (Intercept) | 20 |  |  |  |  |
| Time | 19 | 1 | 9.206 | 0.039 | * |
| Tank | 17 | 2 | 16.877 | 0.009 | ** |
| Time:Tank | 15 | 2 | 9.111 | 0.017 | * |
|  |  |  |  |  |  |
| nsDNA *Darwinella* sp. |  |  |  |  |  |
|  | Res.Df | Df.diff | score | Pr(>score) | Signif. |
| (Intercept) | 20 |  |  |  |  |
| Time | 19 | 1 | 1.129 | 0.852 |  |
| Tank | 17 | 2 | 14.056 | 0.037 | * |
| Time:Tank | 15 | 2 | 4.165 | 0.471 |  |
| Signif. codes: 0 ‘***’ 0.001 ‘**’ 0.01 ‘*’ 0.05 ‘.’ 0.1 ‘ ’ 1 | | | |  |  |

**Appendix S2:**

**Figure S4. Comparing the proportional read counts of each sample and the detectability of each species.** **A.** The linear relationship between the proportional read counts and the time point of each sample type. The points represent sample replicates per tank. The x-axis is the timeline of the sampling events. The “fish present” represents the period when fish were in the tanks, the time represents the time point after fish removal. **B.** The detectability of each fish species per sample type. Symbols represent species. The y-axis is the proportional read counts of each species per sample type, and the x-axis is the total number of positive detections of each fish per sample type. The colours represent the sample type for both A and B.

**Figure S5. Community change over time**. The horizontal panels represent three tanks; the vertical panels show three sample types. Water and sponge samples are separated by the timeline. For each tank, the community changes from top to bottom according to sampling time, and time codes as in Fig. 2A. The length of the bar is proportional read counts. When there is no bar at the corresponding time point, it represents that no fish species were detected. Colour codes for experimental fish species for each tank (three species for each tank).

**Table S6. Linear model output**. Testing for the relationship between proportional read counts and time.

| nsDNA *Darwinella* sp. | |  |  |  |  |
| --- | --- | --- | --- | --- | --- |
|  | Estimate | Std. Error | t value | Pr(>\|t\|) | signif. |
| (Intercept) | 24.947 | 8.829 | 2.826 | 0.0108 | * |
| reads | -16.893 | 18.236 | -0.926 | 0.3659 |  |
| Residual standard error: 29.86 on 19 degrees of freedom | | | | | |
| Multiple R-squared: 0.04321, Adjusted R-squared: -0.007146 | | | | | |
| F-statistic: 0.8581 on 1 and 19 DF, p-value: 0.3659 | | | | | |
|  |  |  |  |  |  |
| nsDNA *Axinyssa* sp. | |  |  |  |  |
|  | Estimate | Std. Error | t value | Pr(>\|t\|) | signif. |
| (Intercept) | 29.486 | 7.455 | 3.955 | 0.000849 | *** |
| reads | -44.3986 | 18.937 | -2.227 | 0.038205 | * |
| Residual standard error: 27.18 on 19 degrees of freedom | | | | | |
| Multiple R-squared: 0.2071, Adjusted R-squared: 0.1653 | | | | | |
| F-statistic: 4.961 on 1 and 19 DF, p-value: 0.0382 | | | | | |
|  |  |  |  |  |  |
| Aquatic eDNA | |  |  |  |  |
|  | Estimate | Std. Error | t value | Pr(>\|t\|) | signif. |
| (Intercept) | 36.41 | 7.09 | 5.136 | 5.87E-05 | *** |
| reads | -41.17 | 11.72 | -3.513 | 0.00232 | ** |
| Residual standard error: 23.77 on 19 degrees of freedom | | | | | |
| Multiple R-squared: 0.3938, Adjusted R-squared: 0.3619 | | | | | |
| F-statistic: 12.34 on 1 and 19 DF, p-value: 0.002323 | | | | | |
|  |  |  |  |  |  |

Signif. codes: 0 ‘***’ 0.001 ‘**’ 0.01 ‘*’ 0.05 ‘.’ 0.1 ‘ ’ 1
